# Molecular basis of ampicillin resistance: combinatorial mechanisms and future strategies

**DOI:** 10.1007/s11274-026-04826-z

**Published:** 2026-02-09

**Authors:** Osman Türkyılmaz, Cihan Darcan

**Affiliations:** 1 Biotechnology Application & Research Centre, Bilecik Seyh Edebali University, Bilecik, Turkey; 2 Department of Molecular Biology and Genetics, Faculty of Science, Bilecik Şeyh Edebali University, Bilecik, Turkey

**Keywords:** Ampicillin, β-lactamase, Mechanisms, Resistance

## Abstract

Due to the increasing antibiotic resistance profile, the efficacy of ampicillin – one of the main treatment options in clinical practice for many years – has markedly declined. Understanding the molecular processes that reduce the effectiveness of this antibiotic is crucial for optimizing current treatment protocols and designing new drugs that are less susceptible to resistance. This review examines the molecular basis of ampicillin resistance from a multilayered perspective, providing a comprehensive analysis of how processes such as β-lactamase production, alterations in penicillin-binding proteins, porin modifications, efflux pump activation, and synergistic combinations of these mechanisms contribute to ampicillin resistance according to current literature. Furthermore, we discuss how different resistance mechanisms collectively establish a coordinated resistance matrix. Finally, we evaluate how these mechanistic insights can inform the development of next-generation therapeutic strategies to overcome ampicillin resistance and assess the future therapeutic potential of multi-targeted approaches.

## Introduction

Ampicillin, which belongs to the aminopenicillins subgroup of β-lactams, is a broad-spectrum antibiotic that can exert a bactericidal effect on both Gram-positive and Gram-negative bacteria (Bereda 2022). Although chemically similar to penicillin, it contains an additional amino group that facilitates its passage through bacterial membranes (Youssif et al. 2023). This antibiotic is widely prescribed to treat various infections, including pneumonia (Yamamoto et al. 2025), urinary tract infections (Vallo et al. 2022), meningitis (Seki et al. 2023), sepsis (Le et al. 2022), enterococcal infections (Suh et al. 2021), and carbapenem-resistant *Acinetobacter baumannii* infections (Bartal et al. 2022). However, its widespread use has promoted the development of resistance mechanisms, reducing the effectiveness of ampicillin and other β-lactam antibiotics. Currently, most pathogens have developed various resistance mechanisms against ampicillin, posing a significant threat to the success of antimicrobial therapy.

Ampicillin resistance has increased and continues to spread rapidly in numerous clinically significant bacterial species, including *Klebsiella pneumoniae* (Al-Baz et al. 2022), *Pseudomonas aeruginosa* (Rezaloo et al. 2022), *Enterococcus* spp. (Iancu et al. 2023), *Enterobacteriaceae* (Mirzaei et al. 2021), *Neisseria meningitidis* (Rostamian et al. 2022), and *Haemophilus influenzae* (Abavisani et al. 2024). Ampicillin resistance can arise through various pathways, including β-lactamase production, changes in penicillin-binding proteins (PBPs), loss of porin channels, and increased efflux pumps. Understanding these resistance mechanisms is crucial for preventing the emerging antibiotic crisis. In this review, we investigate the molecular mechanisms of ampicillin resistance and discuss the combined effect and clinical significance of different resistance mechanisms.

## Mechanism of action of ampicillin

Ampicillins, like many β-lactams, are antibiotics that target PBPs, which are involved in the synthesis of peptidoglycan, a fundamental component of the bacterial cell wall. The bacterial cell wall is composed of glycan chains containing N-acetylglucosamine and N-acetylmuramic acid, as well as pentapeptide bridges that cross-link these chains (Martin et al. 2022). PBPs catalyze the formation of pentapeptide cross-links between peptidoglycan chains through transpeptidation, increasing the mechanical stability of the cell wall. Due to its structural similarity to the D-Ala-D-Ala dipeptide, ampicillin forms a covalent bond by performing a nucleophilic attack on the serine residue in the active site of PBPs (Kim et al. 2023). As a result of ampicillin binding to PBP, PBP is inactivated and transpeptidation does not occur. Maintaining cell wall integrity is vital for the bacterium; however, when the PBP cannot perform its function, the necessary cross-links do not form, weakening the cell wall. A weakened cell wall becomes susceptible to osmotic stress, ultimately resulting in cell autolysis (Mora-Ochomogo and Lohans 2021).

## Mechanisms of ampicillin resistance

### Resistance with β-lactamase enzymes

**Fig. 1:**
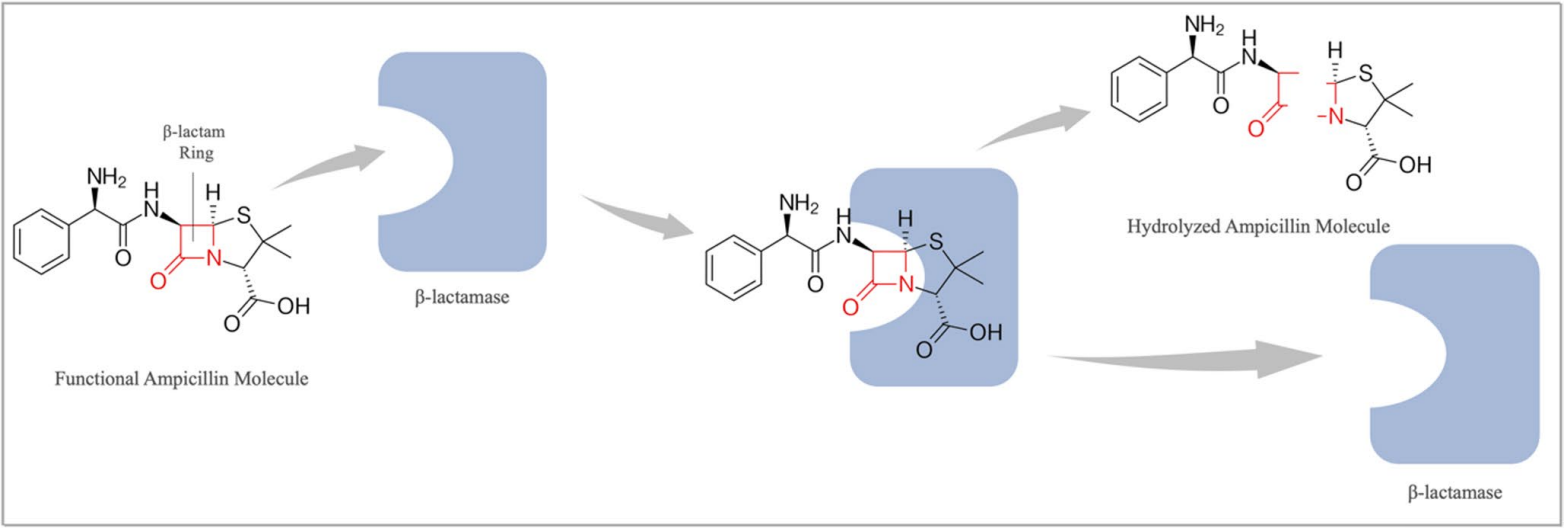
Enzymatic hydrolysis of ampicillin via the β-lactamase

The production of β-lactamases is one of the most common resistance mechanisms used by both Gram-positive and Gram-negative bacteria against β-lactam antibiotics (Alfei and Schito 2022). β-lactamases render antibiotics ineffective by hydrolyzing the β-lactam ring (Fig. 1) (Kaderabkova et al. 2022). β-lactamases are usually encoded on plasmids and transposable elements, allowing organisms to easily share these resistance genes (Zdarska et al. 2024). In addition to their widespread distribution, the continual discovery of new β-lactamases with different capabilities makes combating these enzymes even more difficult.

As of December 2025, more than 12,000 β-lactamases have been identified (Naas et al. 2017). Recent data show that approximately 1000 new β-lactamases have been identified in the past year (Zdarska et al. 2024). With the increasing number of β-lactamases discovered each day, various classification systems have been developed, with the Ambler classification being the most widely used. According to the Ambler classification, β-lactamases are divided into four main groups: classes A, B, C, and D. Classes A, C, and D are also known as serine β-lactamases because they have an active site serine that catalyzes hydrolysis. Class B β-lactamases are known as metallo-β-lactamases because they use zinc ions for their activity. These two groups differ in sequence, structure, and catalytic mechanism (Pandey et al. 2023).

#### Class A β-lactamases

Class A is the most widely distributed group, with more than 2500 identified β-lactamases belonging to this class (Naas et al. 2017). This group includes clinically important enzymes such as TEM, SHV, CTX-M, and KPC (Tooke et al. 2019). The dissemination of these enzymes through plasmids and the expansion of their activity spectrum via mutations pose a significant threat to β-lactam antibiotics. Among Gram-negative bacteria, the most commonly observed plasmid-mediated β-lactamases are TEM enzymes encoded by bla_TEM_, followed by SHV and CTX-M enzymes encoded by bla_SHV_ and bla_CTX−M_, respectively (Elsayed et al. 2024). The KPC enzyme, although more recently emerged than the others, has a broader activity spectrum (Palzkill 2018). A study on *Escherichia coli* isolates found that the presence of bla_TEM_ or bla_CTX−M_ increased ampicillin resistance more than 65-fold compared to the *E. coli* ATCC 25,922 strain, raising it above 500 µg/ml. The presence of both genes together increased ampicillin resistance more than 130-fold, raising it to 1000 µg/ml (Rihacek et al. 2023). Similarly, another study showed that the presence of bla_SHV_ increased ampicillin resistance more than 64-fold compared to wild-type *E. coli* strains, exceeding 1024 µg/ml (Zhu et al. 2017). These MIC values are well above the breakpoints of 8 µg/ml and 32 µg/ml accepted by the European Committee on Antimicrobial Susceptibility Testing (EUCAST) and the Clinical and Laboratory Standards Institute (CLSI) for Enterobacterales, respectively, and indicate that the presence of these enzymes may severely limit the therapeutic efficacy of ampicillin (CLSI 2025; EUCAST 2026). These findings indicate that Class A β-lactamases play a critical role in ampicillin resistance. The coexistence of these enzymes within the same cell significantly reduces the effectiveness of ampicillin. This demonstrates that the spread of class A β-lactamases poses a major threat in hospital infections and community-acquired infectious diseases.

With the emergence of new β-lactam antibiotics, numerous variants of the same enzymes have appeared due to mutations in certain β-lactamases. These mutations can alter the effect of β-lactamases on ampicillin. For example, the G238S and R164S substitutions in TEM-1 β-lactamase increase the hydrolysis rates of cefotaxime and ceftazidime but significantly reduce the hydrolysis rate of ampicillin (Dellus-Gur et al. 2015). Similarly, the A237T substitution in TEM-1 β-lactamase causes a modest increase in cephalosporin resistance but results in decreased hydrolysis of ampicillin (Palzkill 2018). In a study examining amino acid variants at position 104 of the SHV enzyme, 58% of the variants with alterations at this position exhibited reduced resistance to ampicillin while showing increased resistance to cefotaxime (Bethel et al. 2006). In contrast, class A β-lactamase mutations that increase ampicillin resistance have also been identified. Substitution of a cytosine base with adenine at the − 10 region of the bla_SHV_ promoter, or deletion of the downstream region of the promoter, leads to high expression of bla_SHV_ and consequently causes a significant increase in ampicillin resistance (Chen et al. 2012). Another study involving 4,997 variants of TEM-1 β-lactamase showed that mutations in this enzyme can change ampicillin resistance within a range of 10–2500 µg/mL (Stiffler et al. 2015). The clinical resistance breakpoint value for ampicillin has been determined by CLSI as 1–32 µg/mL depending on the organism, while EUCAST has set it at 0.5–8 µg/mL (CLSI 2025; EUCAST 2026). Therefore, most specified TEM-1 β-lactamases frequently exceed the threshold for resistance, rendering standard dose treatment ineffective. These results demonstrate that Class A β-lactamases can be optimized toward specific antibiotics and that resistance mechanisms represent a dynamic process. The effects of mutations in these enzymes on their activities and substrate specificities result in varying levels of ampicillin resistance and the emergence of extended-spectrum β-lactamase variants. This situation requires careful planning of antibiotic use strategies and the development of therapeutic approaches targeting new resistance profiles.

#### Class B β-lactamases

Class B β-lactamases, also known as metallo-β-lactamases (MBLs), are enzymes that primarily confer resistance to carbapenem antibiotics. These enzymes hydrolyze the β-lactam ring using Zn²⁺ ions in their active sites, thereby eliminating the antibiotic’s activity. While many Class B β-lactamases exhibit limited activity against ampicillin, some can efficiently hydrolyze it. Among the clinically significant Class B β-lactamases that confer ampicillin resistance are New Delhi metallo-β-lactamase (NDM), Verona integron-encoded metallo-β-lactamase (VIM), and imipenemase (IMP). Clinical isolates carrying the NDM-1 enzyme are known to exhibit high-level resistance to ampicillin (Xiang et al. 2020). All variants of NDM-1, which is highly stable in terms of resistance, demonstrate approximately an 8,000-fold increase in ampicillin resistance and consistently maintain this elevated resistance. These increases raise the resistance level of microorganisms far beyond clinically achievable ampicillin concentrations (CLSI 2025; EUCAST 2026). Therefore, infections caused by NDM-producing organisms are unlikely to respond to ampicillin treatment. In contrast, VIM subtypes – particularly VIM-2– display a more variable resistance profile against substrates. In a study investigating the impact of amino acid substitutions at different positions of VIM-2 on resistance, most substitutions reduced the level of ampicillin resistance, whereas certain changes at positions 61 and 67 resulted in nearly a two-fold increase in resistance (Borgianni et al. 2010). These findings indicate that the substrate-binding region of VIM-2 has a structurally sensitive effect on enzymatic activity and resistance phenotype. Although IMP-type MBLs confer relatively lower resistance to ampicillin, these levels remain clinically relevant. The activity of IMP variants can vary depending on environmental pressures and the genetic context of their mobile elements. Therefore, all three major MBL types have distinct kinetic and structural properties regarding ampicillin resistance and represent resistance determinants that must be carefully considered in clinical management.

#### Class C β-lactamases

Class C β-lactamases, known as cephalosporinases or AmpC, have long been associated with the gradual loss of efficacy of many antibiotics – including penicillins, cephalosporins, and cephamycins – in treating various bacterial infections (Philippon et al. 2022). Structurally, Class C β-lactamases are similar to penicillin-binding proteins (PBPs), which are the cellular targets of β-lactam antibiotics (Tebano et al. 2024). This structural similarity underlies their interaction with β-lactams. Class C β-lactamases are predominantly encoded on bacterial chromosomes. These chromosomal β-lactamases are widely distributed among Gram-negative bacteria such as *Enterobacter cloacae*, *Citrobacter freundii*, *Serratia marcescens*, *Morganella morganii*, and *P. aeruginosa* (Tamma et al. 2019). However, acquisition of *ampC* genes via plasmids has led to the emergence of these enzymes in species such as *E. coli*, *K. pneumoniae*, and *Proteus mirabilis*, which do not naturally harbor chromosomal *ampC* or express it only weakly. Additionally, mutations in the promoter regions of these genes have been reported to enhance gene expression and result in clinically significant levels of ampicillin resistance. *ampC* promoter mutations most commonly occur within the − 35 box, followed by the spacer region between the − 35 and − 10 boxes, and then the − 10 box itself (Tracz et al. 2007). The most frequently observed mutation within the − 35 box is the T-32 A mutation, which increases gene expression by 10- to 40-fold (Singh et al. 2019). The spacer region typically contains insertion mutations that extend the spacer to an optimal length (Klein et al. 2021). The − 16_–15insG mutation in the spacer region has been shown to increase gene expression by approximately 95-fold (Türkyılmaz and Darcan 2024). Within the − 10 box, the most common mutation is the C–11 A substitution, which elevates gene expression by approximately 20-fold (Singh et al. 2019). In most clinical isolates, more than one of these mutations is present. This allows the organisms to achieve higher levels of β-lactamase expression, thereby attaining higher levels of ampicillin resistance. A strain carrying the T-32 A, C-11 A, −22_−21insT, and 16_43del mutations exhibited a 140-fold increase in *ampC* expression (Tracz et al. 2005). The 220-fold increase in *ampC* gene expression observed in *E. coli* W3310 cells has resulted in the minimum inhibitory concentration for ampicillin exceeding 350 µg/mL (Türkyılmaz and Darcan 2024). This value is well above the 8–32 µg/mL defined as the clinical resistance threshold for *E. coli* (CLSI 2025; EUCAST 2026). This finding demonstrates that AmpC overexpression alone can lead to clinically significant resistance. Therefore, Class C β-lactamases are a significant source of ampicillin resistance. The presence of both chromosomal and plasmid- mediated genes facilitates the spread of this resistance mechanism among bacteria and limits treatment options. Mutations in the promoter region can cause a dramatic increase in gene expression, resulting in serious clinical consequences. The fact that these mutations arise predominantly in combinations, rather than individually, makes the problem even more complex and threatening.

#### Class D β-lactamases

Class D β-lactamases are commonly referred to as oxacillinases (OXA). Although many OXA enzymes are primarily recognized for their ability to hydrolyze carbapenems, they also have intrinsic activity against penicillins such as ampicillin. The early OXA enzymes were initially identified as penicillinases, highlighting the impact of OXA enzymes on ampicillin resistance (Evans and Amyes 2014). In *E. coli* strains carrying OXA-1, the first discovered Class D β-lactamase, the level of ampicillin resistance is four times higher than in strains that do not harbor this enzyme (Livermore et al. 2019). Over time, certain OXA enzymes, such as the OXA-10-derived variants OXA-14 and OXA-16 and the OXA-2-derived OXA-15, have acquired an expanded substrate spectrum due to mutations and have developed resistance to more potent β-lactam antibiotics, including carbapenems (Evans and Amyes 2014). This evolutionary process demonstrates that OXA enzymes, which initially conferred resistance to narrow-spectrum antibiotics such as ampicillin, can develop a broad resistance profile by becoming effective against multiple classes of antibiotics. However, despite these evolutionary changes that have enabled many OXA enzymes to acquire an expanded substrate spectrum, they have not caused a significant change in resistance levels against ampicillin. A rare variant that significantly increases ampicillin resistance has emerged in OXA-51–derived OXA-79. The OXA-79 variant harboring the W222G mutation increases the ampicillin MIC to > 2048 µg/mL, thereby raising resistance more than 64-fold compared to an OXA-51-producing strain and conferring high-level resistance (Chan et al. 2022). The MIC value for the OXA-79 variant is well above the clinical breakpoints defined by CLSI and EUCAST, indicating that this variant is associated with a high level of clinical resistance (CLSI 2025; EUCAST 2026). Therefore, the clinical efficacy of ampicillin will be significantly reduced in the presence of this variant, and the likelihood of treatment failure under standard dosage regimens will be high. These findings show that OXA enzymes can significantly change not only their substrate spectrum but also resistance levels to specific antibiotics through distinct mutations. In particular, as seen with OXA-79, a single mutation can cause a dramatic increase in ampicillin resistance, highlighting the critical evolutionary potential of these enzymes from a clinical perspective. Furthermore, given their low affinity for β-lactamase inhibitors, treatment options for OXA-positive isolates may remain limited, and current combination therapies may not always be effective. Therefore, monitoring OXA variants at the molecular level and thoroughly examining their susceptibility profiles to inhibitors should be among the priority strategies in combating resistance.

### Resistance mediated by penicillin-binding proteins

Penicillin-binding proteins (PBPs) are enzymes responsible for synthesizing the cell wall. The β-lactam ring in ampicillin is a structural analog of the D-alanyl-D-alanine residues at the terminus of the peptidoglycan chain. This molecular similarity underlies the antibiotic’s mechanism of action. When bacteria are exposed to ampicillin, the antibiotic competes with the natural D-alanyl-D-alanine residues and binds irreversibly to PBPs. This binding inhibits the essential transpeptidation function of PBPs, disrupting bacterial cell wall synthesis and leading to cell death (Dabhi et al. 2024). However, microorganisms can counteract the effects of β-lactam antibiotics through various mechanisms.

#### Reduced binding affinity

**Fig. 2:**
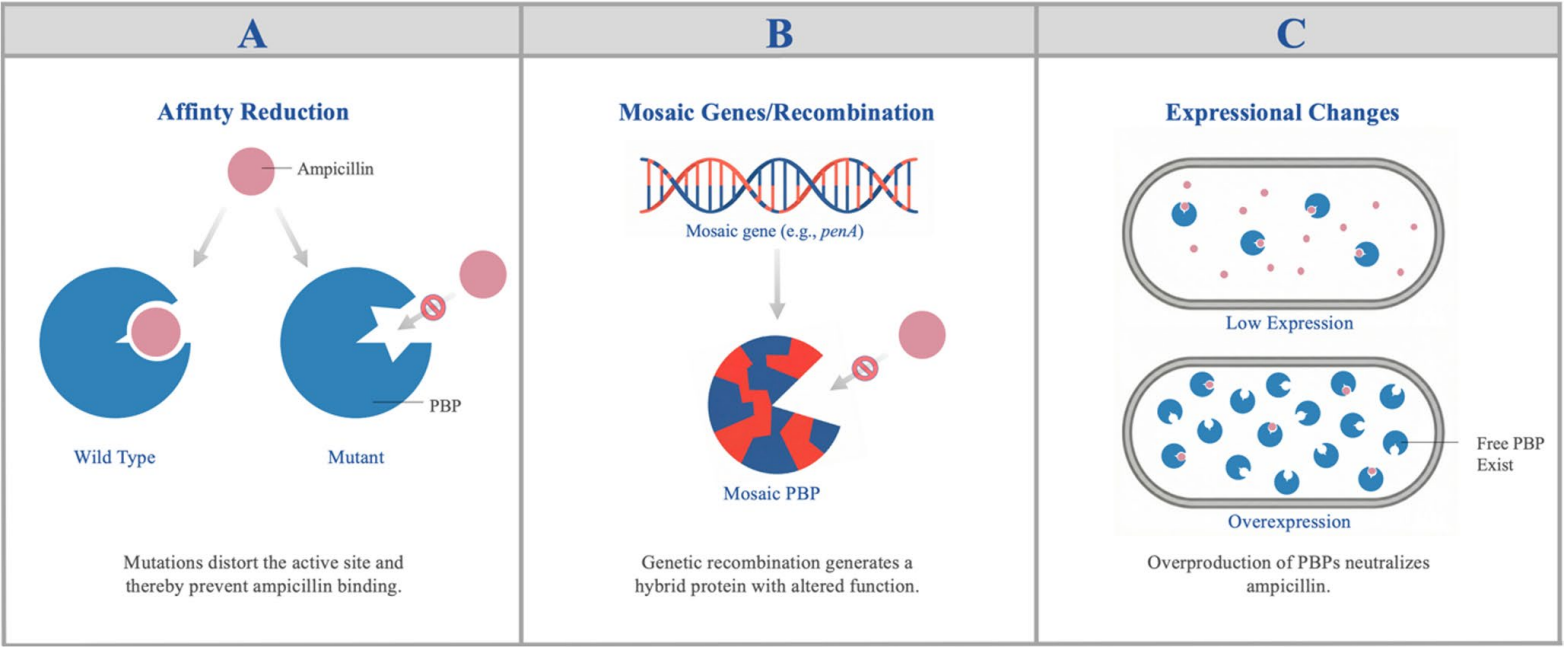
Ampicillin resistance mechanisms mediated by PBPs. (**A**) Reduced Affinity: Point mutations in PBP genes alter the protein’s active site, preventing ampicillin binding. (**B**) Mosaic Genes/Recombination: Genetic recombination produces hybrid (mosaic) PBPs with altered structures and reduced antibiotic affinity. (**C**) Expression Changes: Overproduction of PBPs ensures that, even if ampicillin binds to some proteins, enough free PBPs remain to maintain cell wall synthesis

Point mutations in PBPs can reduce antibiotic affinity, allowing the organism to develop resistance to β-lactams (Fig. 2A). Ampicillin specifically targets FtsI and MrdA in Gram-negative bacteria such as *E. coli*. Ampicillin resistance has arisen through mutations at specific sites within these genes. Amino acid substitutions at positions 536 and 537 of FtsI were identified in various *E. coli* strains passaged in the presence of ampicillin to induce resistance (Xing et al. 2020; Türkyılmaz and Darcan 2024). These findings suggest that amino acids 536 and 537 of FtsI play a significant role in ampicillin resistance. An amino acid substitution at position 389 of MrdA was detected in a different *E. coli* strain exhibiting ampicillin resistance (Thulin et al. 2015). In a study with *H. influenzae*, all nine FtsI variants examined exceeded the 4 µg/mL MIC threshold considered clinically resistant to ampicillin (Jakubu et al. 2024; CLSI 2025). These data indicate that point mutations at specific positions in the PBP targets of ampicillin contribute to resistance by reducing ampicillin affinity. From a clinical perspective, these PBP-mediated resistance mechanisms can cause ampicillin treatment failure even without β-lactamase production. In routine susceptibility testing, which typically assesses only β-lactamase presence, resistance phenotypes linked to PBP variants may be missed, increasing the risk of inappropriate antibiotic selection. Therefore, considering the impact of PBP alterations on MIC levels is an important complementary factor in the clinical management of infections. Detecting these mutations is important for understanding resistance mechanisms and guiding treatment strategies.

#### Mosaic genes/recombination

DNA fragments taken up by bacteria from their environment can recombine with their own genes, forming mosaic gene sequences. Such recombination events also occur in PBP genes. Mosaic PBP proteins produced through this process may have reduced ampicillin affinity and increased resistance levels (Fig. 2B) (Kobras et al. 2023). In mosaic PBP genes, the binding sites for ampicillin and other β-lactams are altered, involving numerous amino acid variants. As a result, resistance levels can be much higher than those caused by single-base changes. The *penA* gene encodes PBP2 in *N. meningitidis*. Mosaicism in *penA* has led to a moderate increase in resistance to ampicillin and penicillin antibiotics (Potts et al. 2022). These types of mosaic PBP structures often result in gradual but clinically significant increases in MIC values. In isolates with mosaic PBP genes, these increases may be reported as borderline or moderate resistance in standard susceptibility tests but can reduce the effectiveness of β-lactam therapy (Zapun et al. 2008). The emergence of such mosaic structures occurs in a geography and clone specific manner (Mousavi et al. 2017). This suggests the existence of different mutational and evolutionary pathways. Such diversity is important for understanding the global epidemiology of antibiotic resistance and for developing new therapeutic strategies.

#### Expressional changes

Besides structural alterations in PBPs, increased PBP expression can also elevate ampicillin resistance. When the amount of PBP proteins with low affinity for antibiotics rises, the antibiotic cannot inhibit all of these molecules, allowing bacterial cell wall synthesis to continue uninterrupted (Fig. 2C) (Montealegre et al. 2016). *Enterococcus faecium*, a common cause of hospital-acquired infections, naturally possesses PBP5, which has reduced affinity for ampicillin. In a study of *E. faecium* clinical isolates, various variations were identified in the C-terminal region of *pbp5*. Additionally, PBP5 levels were significantly higher compared to susceptible strains, and there was a positive correlation between PBP5 levels and ampicillin MIC values (Darehkordi et al. 2019). This indicates that mutations alone cannot fully explain ampicillin resistance and that expression levels also play a role. *psr* alleles play an important role in regulating PBP5 expression (Singh et al. 2024). These alleles repress PBP5 expression; however, some psr variants lack this repressive function. Consequently, strains carrying these psr variants are associated with high PBP5 expression and increased resistance to ampicillin (Singh et al. 2024). These findings demonstrate that different psr variants can have distinct effects on PBP5 expression. In conclusion, it has been demonstrated that not only structural mutations but also alleles regulating gene expression play a role in ampicillin resistance. This underscores the need to consider gene expression levels, in addition to mutations, when analyzing resistance mechanisms.

### Resistance mediated by porin alterations and efflux pumps

In the development of ampicillin resistance in Gram-negative bacteria, the alterations of porin channels that restrict antibiotic entry through the outer membrane and the action of efflux pumps that actively expel toxic compounds play a significant role. These two mechanisms work synergistically to keep the antibiotic concentration within the cell below a critical level (Li et al. 2015; Bartsch et al. 2021).

#### Downregulation and modification of porins

**Fig. 3:**
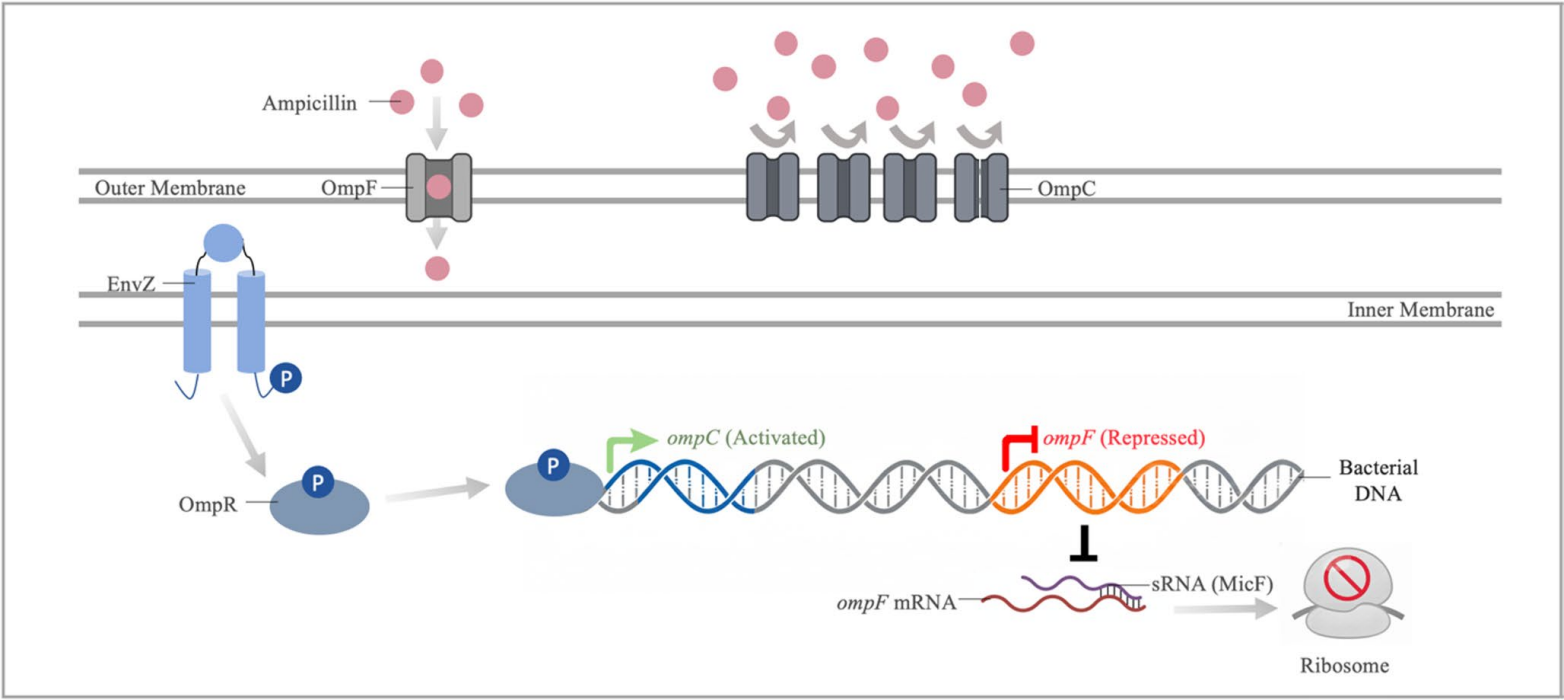
Ampicillin resistance mechanism associated with porin regulation. The diagram shows changes in the expression of porin-encoding genes (*ompC* and *ompF*) resulting from the interaction between EnvZ and OmpR proteins. Due to OmpR-P-mediated transcriptional regulation and MicF-mediated post-transcriptional repression, the level of OmpF in the outer membrane decreases, while the level of OmpC increases. This change in porins reduces the uptake of antibiotics such as ampicillin into the cell

Outer membrane permeability is fundamental to the entry of many antibiotics into cells. However, bacteria can limit this permeability by reducing porin gene expression under stress or through mutations in porin structure. This is an important mechanism in the development of antibiotic-resistant phenotypes. Porin expression is directly influenced by environmental signals. Under environmental stress and antibiotic pressure, regulatory mechanisms of porin genes are activated, reducing outer membrane permeability (Ko and Choi 2022; Zhou et al. 2023). The main factors responsible for this include two-component systems and global stress proteins. Regulation of OmpF and OmpC porin expression is a critical adaptation affecting permeability in ampicillin resistance (Fernández and Hancock 2012). Central to this regulation is the EnvZ/OmpR two-component system. EnvZ acts as a sensor that detects environmental conditions and phosphorylates OmpR, the cytoplasmic regulator. Phosphorylated OmpR, binds to the promoters of *ompF* and *ompC* and regulates their expression (Gerken et al. 2020). Under stress conditions, the EnvZ/OmpR system typically increases *ompC* expression, which has a narrower channel, while decreasing *ompF* expression, which provides a wider entry point for ampicillin (Fig. 3). OmpC is narrower than OmpF, it physiologically restricts ampicillin uptake. Analysis of outer membrane proteins in an *E. coli* K-12 strain with 32-fold ampicillin resistance revealed a marked increase in OmpC porin protein expression compared to the control strain (Xu et al. 2006). A 15-fold decrease in *ompF* expression was observed in the *E. coli* W3110 strain exhibiting 10-fold ampicillin resistance (Türkyılmaz and Darcan 2024). These findings demonstrate that increased OmpC expression and decreased OmpF expression are important mechanisms for survival in the presence of ampicillin and other β-lactams. Following these changes in the porin profile, it is considered that the reported ampicillin MIC values may exceed the clinical susceptibility thresholds defined by CLSI and EUCAST, which could increase the risk of treatment failure (CLSI 2025; EUCAST 2026).

Under environmental stress conditions, global regulators indirectly influence porin expression through sRNAs. sRNAs such as MicF and MicC can inhibit ribosomal activity via post-transcriptional control. MicF binds to the region of *ompF* mRNA that includes the ribosome-binding site and start codon, thereby inhibiting the translation of OmpF (Fig. 3) (Tyulenev et al. 2022). MicC, on the other hand, directly binds to the 5’ UTR of *ompC* mRNA and inhibits OmpC translation (Doranga and Conway 2023). The global regulator MarA contributes to ampicillin resistance by reducing the expression of the OmpF porin through MicF (Dam et al. 2018). In ampicillin adaptive resistance studies, promoter mutations in *marA* emerging in the early stages increased *marA* expression by approximately 20-fold, and in parallel, *ompF* expression decreased by about 15-fold (Türkyılmaz and Darcan 2024). This clearly demonstrates the role of MarA in ampicillin resistance. Another sRNA known to contribute to ampicillin resistance is sRNA1039, found in *Shigella sonnei*. sRNA1039 acts as a post-transcriptional regulator that increases the stability of cfa mRNA. cfa expression represses the expression of *ompD*, a porin gene, thereby reducing the number of porins in the cell membrane and contributing to ampicillin resistance (Du et al. 2024).

Antibiotic uptake can be reduced or inhibited not only by changes in gene expression but also by mutations that alter porin structure. Therefore, bacteria can also develop resistance to antibiotics in this way. These mutations are generally adaptive changes that decrease antibiotic uptake without impairing channel function. This demonstrates that bacteria have strong adaptive flexibility in response to stress factors. Mutations in porins can cause channel narrowing, changes in charge distribution, and large-scale structural disruptions. The region within the porin that directly controls permeability is the L3 loop, which forms the narrowest part of the channel (Vasan et al. 2022). Mutations are typically located in the L3 loop region and lead to a significant restriction of the space within the porin and/or a change in the electrostatic field, which restricts the passage of charged molecules such as β-lactams. For example, the G112D substitution in Omp36 of *Klebsiella aerogenes* and the G119D substitution in OmpF of *E. coli* significantly reduce the channel diameter and alter the electrostatic field, leading to resistance to all β-lactam antibiotics, including ampicillin (Davin-Regli et al. 2024). A similar situation has been observed in *N. meningitidis* with the PorB porin. A single point mutation resulting in the G103K substitution in PorB changed the geometry and electrostatic properties of the L3 loop, markedly reducing antibiotic uptake. As a result, ampicillin permeation decreased, leading to resistance to ampicillin (Bartsch et al. 2021). Such structural changes may increase MIC values and reduce the clinical response to β-lactam therapy. Changes in porin structure limit antibiotic entry into cells, reducing the effective drug concentration at target sites and negatively affecting pharmacodynamic efficacy. This mechanism can render standard β-lactam treatments inadequate, particularly in severe infections.

For ampicillin to exert its activity, it is sufficient for it to be transported into the periplasmic space. Since the only barrier between the periplasmic space and ampicillin is the outer membrane, outer membrane permeability plays a crucial role in the development of ampicillin resistance. The expression of porins is tightly regulated under environmental stress and antibiotic pressure through two-component systems, global regulators, and sRNAs. Changes in the expression of porins such as OmpF and OmpC are a critical mechanism by which bacteria develop resistance to β-lactam antibiotics. In addition, mutations in porins lead to alterations in their structure and electrostatic properties, thereby restricting antibiotic passage and contributing to resistance. These findings demonstrate that antibiotic resistance mechanisms are complex and multilayered at both the gene expression and structural levels, and highlight that targeting antibiotic uptake mechanisms is an important component of efforts to combat resistance.

#### Overexpression of efflux pumps

Another mechanism as important as preventing the entry of antibiotics into the cell is the efflux of antibiotics out of the cell. Through this mechanism, the intracellular concentration of antibiotics is directly reduced (Nishino et al. 2021). Multidrug efflux pumps play a significant role in the emergence of antibiotic resistance in bacterial pathogens. Different efflux pumps exert varying levels of influence on antibiotic resistance; while the activity of some efflux pumps confers high levels of resistance, others provide low levels of resistance (Chang et al. 2022). Bacteria possess many different types of efflux pumps, which are divided into five major families classified according to structure and energy source: Major Facilitator Superfamily (MFS), Small Multidrug Resistance (SMR), Multidrug and Toxin Extrusion (MATE), ATP-Binding Cassette (ABC), and Resistance-Nodulation-Division (RND) (Reygaert 2018).

All bacteria possess efflux pumps, but Gram-negative bacteria in particular use this mechanism as an important tool for antibiotic resistance. In Gram-positive bacteria, efflux pumps are generally encoded on the chromosome, although a small number of plasmid-borne efflux pumps have also been identified. These pumps typically belong to the MATE and MFS families (Sinha et al. 2024; Brdová et al. 2024; Rajput et al. 2024). Although efflux pumps from all families are present in Gram-negative bacteria, members of the RND family are associated with the highest levels of clinical resistance (Alav et al. 2021). RND pumps can export a wide range of structurally and chemically diverse antibiotics, including ampicillin. Overexpression of these pumps reduces the intracellular accumulation of antibiotics, thereby inhibiting their effectiveness (Ebbensgaard et al. 2020).

**Fig. 4:**
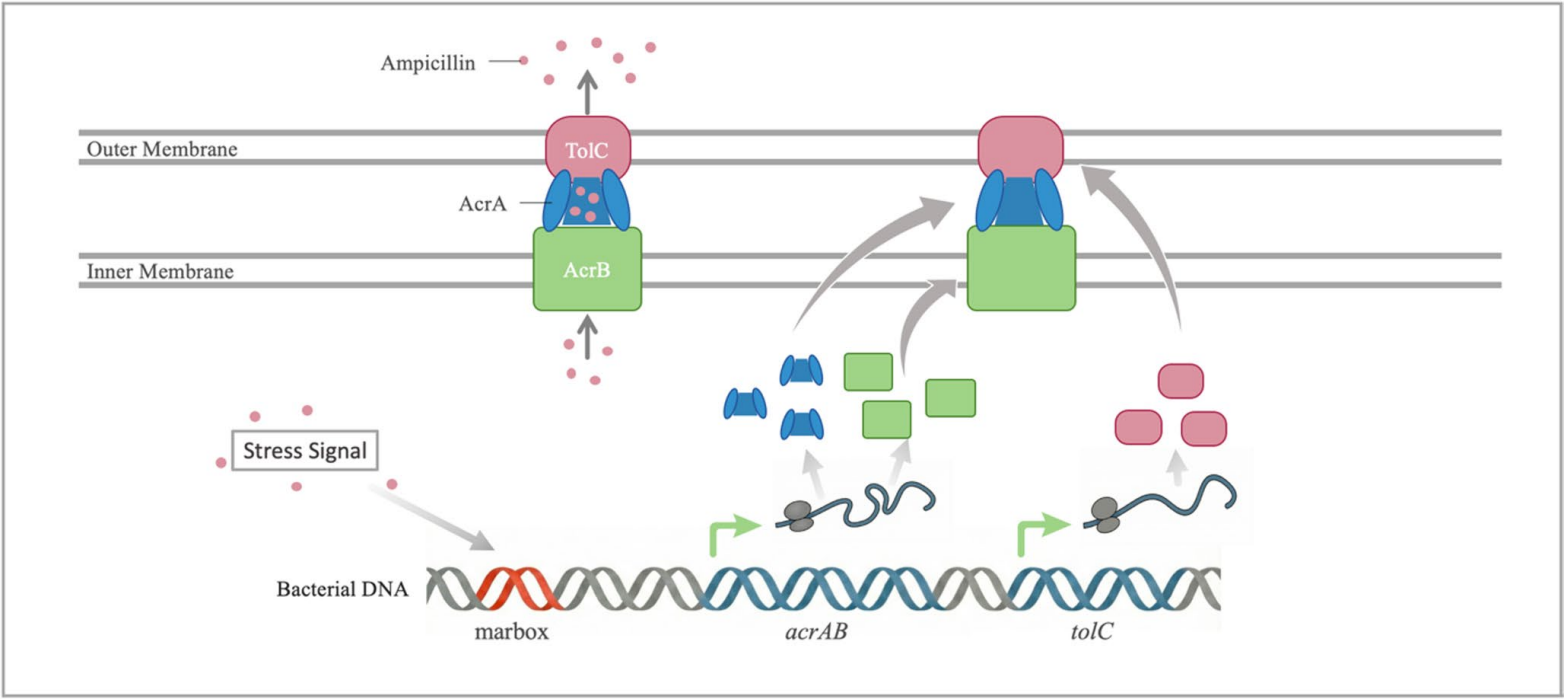
Schematic representation of the transcriptional regulation of the AcrAB-TolC efflux pump system and the ampicillin resistance mechanism. Stress signals activate the marbox regulatory region on the bacterial DNA, triggering the expression of the *acrAB* and *tolC* genes. The synthesized AcrB (inner membrane transporter), AcrA (periplasmic adaptor protein), and TolC (outer membrane channel) assemble to form the functional efflux pump. This tripartite complex actively expels ampicillin molecules out of the cell, conferring bacterial resistance

The AcrAB–TolC complex, an RND efflux system commonly found in Gram-negative Enterobacterales, expels ampicillin and many β-lactam antibiotics from the cell. This complex is composed of AcrB, an inner membrane transporter; AcrA, a periplasmic bridging protein; and TolC, an outer membrane channel (Fig. 4) (Jang 2023). In terms of molecular structure and size, ampicillin is smaller and more polar than antibiotics such as rifampicin. This enables ampicillin to readily bind to the multiple binding pockets of AcrB and be efficiently expelled (Nishino et al. 2021). Thus, the AcrAB–TolC complex is a key resistance mechanism that maintains the intracellular concentration of ampicillin below a critical threshold. Increased expression of the *acrAB* operon and *tolC* has led to resistance to a wide range of antibiotics, including ampicillin (Smith et al. 2024). In a study with *E. coli*, the initial MIC for ampicillin was 2 µg/ml in a susceptible strain lacking the *acrAB* genes. When these genes were introduced and expressed via a plasmid, the MIC increased eightfold to 16 µg/ml. According to the clinical breakpoint, this increase places the strain in the intermediate category between susceptible and resistant thresholds by CLSI standards, while under EUCAST standards, it exceeds the resistance threshold of 8 µg/ml and is classified as resistant (CLSI 2025; EUCAST 2026). Clinically, this shows that expression of efflux pumps such as AcrAB-TolC alone can render standard antibiotic treatments ineffective.

Transcription activators such as MarA, SoxS, and Rob bind to a region called marbox in *E. coli* and control the expression of the *acrAB* and *tolC* genes (Fig. 4) (Wright et al. 2025). Overexpression of any of these activators significantly increases antibiotic resistance in *E. coli* (Bahaj et al. 2025). Environmental signals such as oxidative stress, exposure to organic solvents, or low-level antibiotic pressure can induce the expression of these regulatory proteins, leading to overproduction of the AcrAB–TolC complex (Chetri et al. 2020). This results in resistance not only to ampicillin but also to many structurally diverse antibiotics, disinfectants, and toxic compounds. Therefore, the AcrAB–TolC system is considered one of the key determinants of multidrug resistance. Moreover, efforts to inhibit this system are important for developing new therapeutic strategies that may restore antibiotic effectiveness.

### Combinational effect of resistance mechanisms

**Fig. 5:**
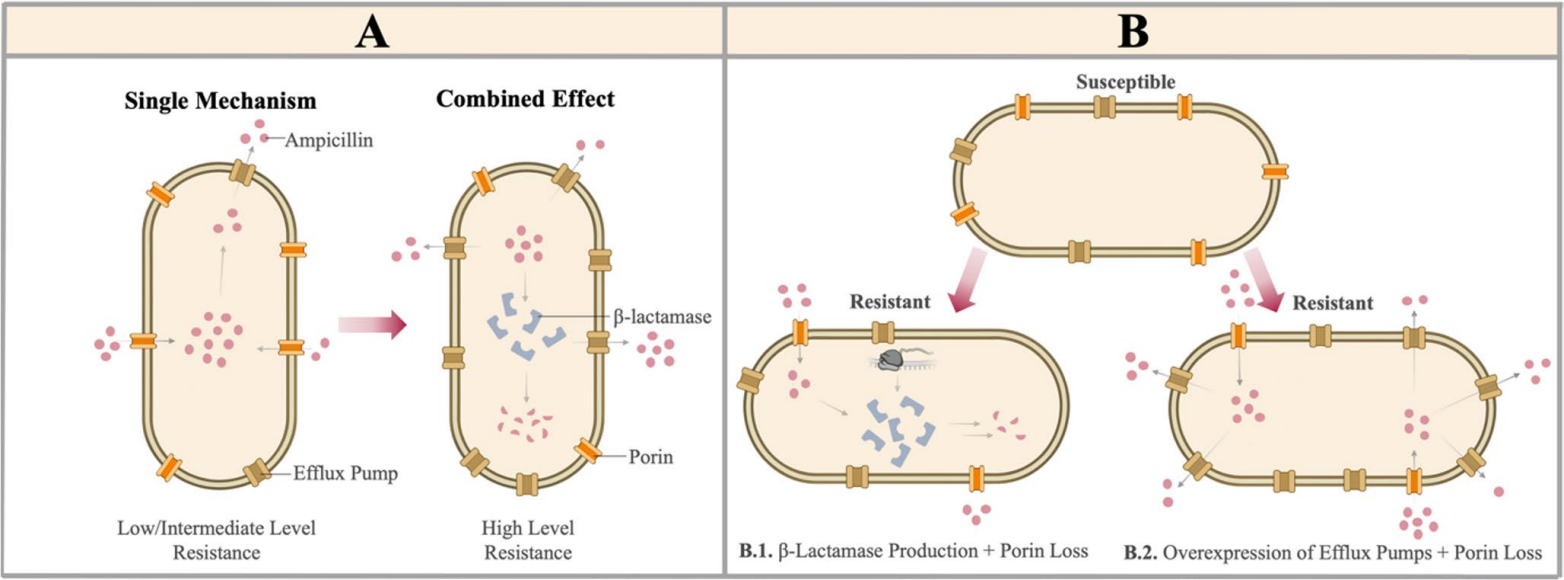
The combined effect of multiple resistance mechanisms on ampicillin resistance. (**A**) Comparison of single and combined resistance mechanisms. (**B**) A susceptible bacterial cell (top) becomes resistant through different combinations of resistance mechanisms. (**B.1**) Porin loss occurring simultaneously with β-lactamase production both restricts antibiotic entry and causes its inactivation. (**B.2**) The combination of overexpression of efflux pumps and porin loss reduces intracellular drug concentration, thereby conferring resistance

Combinatorial effects refer to the simultaneous activation of multiple resistance mechanisms by the bacterium, which synergistically reduce antibiotic efficacy. A single resistance mechanism often does not confer a high level of resistance to an antibiotic. Clinically, the most challenging resistance phenotypes occur when multiple mechanisms function together. The concurrent activation of systems such as β-lactamase production, target protein alterations, reduced permeability, and efflux systems creates a synergistic resistance profile for ampicillin (Fig. 5A). This may offset the fitness cost for the bacterium and enable the development of a more stable resistance phenotype under environmental antibiotic pressure (Li et al. 2022).

Combinatorial effects may be observed differently across cells. One example is the coexistence of β-lactamase production and porin loss within the same cells. In a study of clinical isolates of *E. coli* and *K. pneumoniae*, it was shown that when β-lactamase production occurred together with porin loss or modification, β-lactam resistance reached high levels (Fig. 5B) (Khalifa et al. 2021). In a study on *K. pneumoniae*, strains producing only SHV-type β-lactamase were compared with strains producing SHV-type β-lactamase and exhibiting porin loss. The results showed that MIC values for ampicillin in strains with both resistance mechanisms were more than double those in strains producing only SHV-type β-lactamase, exceeding 512 µg/mL (Chevalier et al. 2000). These resistance levels are well above the ampicillin resistance thresholds defined by both CLSI and EUCAST, indicating that ampicillin is clinically ineffective against these strains (CLSI 2025; EUCAST 2026). This combinatorial effect results from both reduced antibiotic entry into the cell and enzymatic degradation of the antibiotic that does enter. Another example of a combinatorial effect is the simultaneous occurrence of porin loss or modification with efflux pump activity (Fig. 5B). These two mechanisms often act together against antibiotics. In a study of *E. coli* isolates from secondary infections in COVID-19 patients, all isolates were resistant to ampicillin, and in a subset of these strains, porin loss and efflux pumps were shown to function in combination (Ganjo et al. 2024). In a separate study investigating the contribution of porin loss and efflux pumps to antibiotic resistance, *P. aeruginosa* strains with porin loss, active efflux pumps, and both resistance mechanisms simultaneously were compared. The results showed that the MIC value for ampicillin was 64 µg/mL in strains exhibiting only porin loss, 16 µg/mL in strains with only efflux pump activity, and 256 µg/mL in strains with both mechanisms (Krishnamoorthy et al. 2017). These findings indicate that the coexistence of porin loss and efflux pump activity leads to a significant and synergistic increase in resistance to ampicillin.

Adaptive laboratory evolution experiments play a critical role in understanding how combinatorial effects arise by systematically tracking how microorganisms adapt over evolutionary time under defined environmental pressures (Türkyılmaz and Darcan 2023). Mutations in the *ftsI* and *marR* genes have been identified in *E. coli* strains during the early stages of ampicillin adaptation, conferring low-level ampicillin resistance (Li et al. 2019; Türkyılmaz and Darcan 2024). This suggests that PBP modification and marR-mediated changes in porin expression occur during the initial phase of ampicillin resistance. The combined effect observed at low resistance levels causes the MIC value against ampicillin to increase by approximately tenfold to 25 µg/ml, while other resistance mechanisms also contribute to higher resistance levels as the adaptation process continues. *E. coli* strains with high resistance levels, in addition to the previously mentioned mutations, mutations in the *ampC* promoter region (located within the *frdD* gene) enabled *ampC* expression, adding β-lactamase activity to the combined effect. The MIC value increased by approximately 95-fold to 275 µg/ml with the addition of *ampC* β-lactamase activity to the combined effect. (Türkyılmaz and Darcan 2024). The fact that mutations with relatively modest initial effects later contribute to much stronger adaptive responses when additional mechanisms are acquired demonstrates that resistance evolution is not linear but stepwise. This modular structure highlights the importance of epistatic interactions, in which early evolutionary steps lay the groundwork for subsequent adaptations. Thus, the observed integration of multiple sequential mechanisms not only explains why the combinatorial effect is critical for understanding the evolutionary dynamics of antibiotic resistance, but also shows that adaptive evolution experiments are indispensable for forecasting future resistance and developing counterstrategies.

Combinatorial effects can occur not only between different mechanisms but also within a single mechanism. For example, multiple PBP-related mechanisms can combine within the same bacterium to produce stronger resistance. In methicillin-resistant *Staphylococcus aureus* (MRSA) strains, the acquisition of a new low-affinity PBP, PBP2a, along with mutations in the regulatory elements (MecI/MecR1) controlling its expression, results in broad resistance to nearly all β-lactam antibiotics, including ampicillin. Another example is found in *Streptococcus pneumoniae*, where mutations in the PBP2x protein have combinatorial effects that increase resistance levels (Zapun et al. 2008). A similar situation is observed with mutations that alter β-lactamase expression. *E. coli* W3110 strain, the ampC promoter is structurally a weak promoter; therefore, transcription of the *ampC* gene is very low or absent. However, mutations within the promoter region can increase RNA polymerase binding efficiency and thus elevate *ampC* expression (Singh et al. 2020). A single mutation in this region can increase gene expression up to 50-fold, depending on its position and type (Türkyılmaz and Darcan 2024). β-lactamase–producing clinical isolates harbor many additional mutations in this region, the combined effects of which increase *ampC* expression more than 140-fold (Tracz et al. 2005). Given that a 220-fold increase in *ampC* expression, achieved through multiple plasmid copies in *E. coli* W3310 cells, raised the ampicillin MIC above 350 µg/mL, it is clear that combined mutations increasing *ampC* expression are highly important for clinical treatment failure (Türkyılmaz and Darcan 2024). These findings demonstrate that combinatorial effects are not merely the sum of independent factors; rather, a multi-layered synergy can arise even within the same resistance mechanism.

Recent genomic analyses have shown that these multiple mechanisms can be co-carried via mobile genetic elements. Plasmids, transposons, and integrons can simultaneously carry β-lactamase genes, porin regulator genes, or efflux pump activators (Tseng et al. 2023; Wang et al. 2023). This situation demonstrates that combined antibiotic resistance can be acquired rapidly and in a coordinated manner not only through the accumulation of individual mutations, but also through the horizontal transfer of genetic material. Gene clusters carried together by mobile genetic elements provide bacteria with “pre-assembled combinations,” enabling the simultaneous maintenance and dissemination of combinatorial resistance mechanisms. This facilitates the rapid spread of resistance profiles within individual bacteria and across populations and ecosystems, thereby promoting the emergence of MDR strains. Furthermore, this mechanism indicates that antibiotic treatment strategies focusing solely on a single drug may be insufficient, as different resistance factors can be co-selected. Therefore, combinatorial resistance is a multidimensional phenomenon that must be considered in both clinical treatment planning and public health policies. Adaptive evolution experiments and genomic analyses are indispensable tools for understanding these complex interactions and their potential for dissemination.

### SOS-mediated regulation of ampicillin resistance

Antibiotic exposure not only activates known resistance mechanisms but can also trigger the cellular stress response, thereby promoting the acquisition of these resistance mechanisms. The SOS response, a cellular stress response, is a highly conserved defence mechanism in both Gram-negative and Gram-positive bacteria that controls the expression of numerous stress and repair genes. This mechanism sometimes supports DNA repair, attempting to preserve the genetic integrity of the organism, while in other cases it increases the expression of error-prone DNA polymerases, which can raise the likelihood of mutations (Crane et al. 2021). Therefore, exposure to an antibiotic such as ampicillin can trigger the emergence of different resistance mechanisms in the cell via the SOS response (Türkyılmaz and Darcan 2023).

It is known that the SOS response is closely related to horizontal gene transfer (Hocquet et al. 2012). The SOS response triggered by ampicillin exposure may promote the replication and high-frequency horizontal transfer of virulence factors encoded in pathogenicity islands. A study with *S. aureus* found that subinhibitory concentrations of ampicillin induced a true SOS response, characterised by activation of RecA and LexA proteins, leading to increased horizontal transfer of virulence genes (Maiques et al. 2006). Another study showed that ampicillin increased the conjugative transfer of ESBL plasmids in different *E. coli* strains (Liu et al. 2019). This indicates that ampicillin stress triggers horizontal gene transfer mechanisms such as conjugation and transduction, facilitating the spread of resistance and virulence factors. Therefore, the SOS response that arises under ampicillin pressure facilitates the acquisition and dissemination of resistance genes via horizontal gene transfer. This suggests that subinhibitory antibiotic concentrations may contribute not only to treatment failure but also to the enhancement of resistance and virulence traits at the population level.

Another mechanism that increases survival via the SOS response during ampicillin exposure involves the *dpiAB* two-component system. This system forms an operon that encodes the *dpiB* (sensor kinase) and *dpiA* (response regulator) genes, enabling the bacterium to adapt to environmental stresses (Kang et al. 2022). PBP inactivation resulting from ampicillin exposure activates the DpiBA two-component signal transduction system. The active DpiA functions not only as a transcriptional regulator but also as a unique protein capable of binding to A + T-rich regions at chromosomal replication origins, and it inhibits the initiation of DNA replication by binding to *oriC* (Mandin and Gottesman 2009). This leads to activation of the SOS response and increased expression of the *sfiA* (*sulA*) gene, a cell division inhibitor. Increased SfiA protein binds to FtsZ, preventing septum formation and temporarily halting cell division (Burby and Simmons 2020). Therefore, protection against the antibiotic ampicillin, which acts only on actively dividing cells, can be achieved. This mechanism does not provide permanent resistance but serves as a temporary defence that eliminates the antibiotic’s lethal effect for a limited time.

β-lactams significantly disrupt metabolic balance in cells and induce oxidative stress, in addition to inhibiting cell wall synthesis. These antibiotics affect the respiratory chain and tricarboxylic acid cycle, disrupting the cellular electron transport chain and increasing the production of reactive oxygen species (ROS) (Ye et al. 2024). Increased ROS accumulation in the cell can damage DNA, proteins, and lipids, leading to further disruption of cellular balance. Under these oxidative stress conditions, DNA strand breaks occur, triggering activation of the SOS response. During this response, RecA, the regulator of the SOS response, promotes the degradation of LexA, a transcriptional repressor, thereby allowing the expression of SOS genes normally suppressed by LexA. Once LexA repression is lifted, genes involved in DNA repair and replication are activated in the early stages, while in the later stages, DNA damage tolerance genes such as pro-mutagenic translesion synthesis DNA polymerases are activated (Cory et al. 2024). This situation allows replication to continue by bypassing damaged regions where DNA polymerase is stalled, but it also increases the mutation rate. As a result, antibiotic-resistant variants may emerge due to these mutations at the end of this process. Thus, ROS-induced DNA damage and the subsequent SOS response in bacteria exposed to ampicillin and other β-lactams create adaptive evolutionary pressure that increases cellular tolerance in the short term but accelerates the development of resistance in the long term.

These findings show that ampicillin and other β-lactam antibiotics trigger an adaptive stress response that extends well beyond the inhibition of cell wall synthesis. The bacterial response includes short-term tolerance mechanisms such as arresting cell division and activating DNA repair, while long-term mechanisms involve acquiring permanent resistance through mutagenesis and the spread of mobile genetic resistance elements. The observation of these responses even at subinhibitory concentrations underscores the significance of this mechanism in clinical and environmental contexts. Re-evaluating strategies for the use of β-lactam antibiotics to consider not only their target specificity but also the cellular stress responses they induce and their evolutionary consequences will support the development of new, more comprehensive approaches to combating antibiotic resistance.

## Conclusions and future perspectives

Ampicillin resistance is a complex, multifactorial, and dynamic process that cannot be explained by a single biochemical mechanism. The data presented in this review suggest that a single mechanism may contribute to low-level ampicillin resistance, but achieving higher levels of resistance requires the organization of independent mechanisms in a hierarchical, sequential manner, often with strong epistatic interactions. This indicates that resistance mechanisms with combinatorial effects underlie the high-level resistance profiles observed in clinical isolates and that these mechanisms also reshape evolutionary fitness capacity.

Current research directions and strategies for combating ampicillin resistance remain inadequate. The multilayered and synergistic nature of resistance shows that it is controlled by an integrated network that simultaneously activates antibiotic hydrolysis, prevents binding to the target, restricts entry into the cell, and promotes active efflux from the cell. Therefore, future therapeutic approaches should be designed to disrupt the overall functioning of this network rather than targeting individual resistance mechanisms. The rational design of multi-targeted treatment protocols combining β-lactamase inhibitors, efflux pump inhibitors, and porin permeability-enhancing molecules appears to be one of the most promising strategies for overcoming high-level resistance. This combinatorial approach enables the parallel blockade of mutually reinforcing mechanisms such as enzymatic hydrolysis and reduced permeability.

Adaptive laboratory evolution studies are important tools for generating clinical foresight by revealing the gradual and modular structure of resistance. Identifying the mutational pathways followed by bacterial species under selective antibiotic pressure can provide significant information on how treatment processes can be optimized in the early stages of resistance development. Identifying mutations responsible for resistance and mapping the synergistic effects of mutation combinations in antibiotic resistance can lead to a better understanding of the multilayered structure of resistance and early identification of the most risky evolutionary pathways. As a result, predicting the extent of resistance development can improve antibiotic development and treatment processes.

One of the greatest challenges in combating antibiotic resistance is the lack of models that can predict the development of resistance. Although existing data show that certain mutational pathways are similarly selected during resistance development, the parameters determining the selection of these pathways are not fully understood. In the future, predictive maps of resistance development may be generated by integrating adaptive laboratory experiments, genomic data, and computational evolution models. These maps could enable a revolutionary transformation in the design and development of antibiotics used to fight microorganisms.

One of the most critical research areas for the future is emerging in bacterial membrane biology. The regulation of membrane permeability through porins in Gram-negative bacteria highlights the membrane’s role in the development of antibiotic resistance. The dynamic regulation of membrane permeability suggests that many factors may contribute to resistance. Therefore, membrane fluidity, lipid composition, folding kinetics of outer membrane proteins, and the interaction of porin expression with environmental stress can be analyzed using computational biology approaches. This information may lead to the identification of new parameters in antibiotic design.

Efflux pumps, besides exporting antibiotics, are multifunctional structures involved in maintaining metabolic homeostasis, membrane potential, and cellular redox balance. Thus, discovering methods to inhibit efflux pump function offers a powerful strategy to enhance treatment efficacy by affecting the bacterium’s overall physiology. However, developing clinically applicable efflux inhibitors requires a more detailed understanding of pump conformations, their broad substrate range, and their energy utilisation mechanisms. Therefore, generating time-resolved molecular dynamics models for the characterisation and analysis of efflux pumps may mark the beginning of a new era in inhibitor design.

Resistance mechanisms, like many other cellular processes, rely on regulatory control. Therefore, targeting transcriptional and post-transcriptional regulation is a highly important research focus in antibiotic resistance studies. Findings indicate that targeting regulatory elements such as sRNAs, global transcription factors, and two-component systems may allow intervention in the early stages of resistance and prevent bacterial populations from developing a high-level resistance phenotype. Thus, pharmacological modulation of regulators may offer a new strategy in the fight against antibiotic resistance.

Finally, monitoring and identifying mobile genetic elements such as plasmids, integrons, and transposons that simultaneously carry multiple resistance mechanisms has become essential. These elements accelerate the spread of combined resistance mechanisms by carrying not only β-lactamase genes but also porin regulatory genes, efflux activators, and PBP modification genes. Therefore, the evolutionary origins, transfer rates, host compatibility, and dissemination dynamics of combined resistance clusters within mobile genetic elements should be central to future research. Combating antimicrobial resistance requires the development of approaches that monitor not only the genetic structure of individual bacteria but also the circulation of resistance genes across ecosystems.

## Data Availability

No datasets were generated or analysed during the current study.
